# Dragonfly Neurons Selectively Attend to Targets Within Natural Scenes

**DOI:** 10.3389/fncel.2022.857071

**Published:** 2022-04-05

**Authors:** Bernard John Essex Evans, David Charles O’Carroll, Joseph Mahandas Fabian, Steven D. Wiederman

**Affiliations:** ^1^School of Biomedicine, The University of Adelaide, Adelaide, SA, Australia; ^2^Department of Biology, Lund University, Lund, Sweden

**Keywords:** dragonfly (Anisoptera), vision science, electrophysiology, natural images (NI), selective attention

## Abstract

Aerial predators, such as the dragonfly, determine the position and movement of their prey even when both are moving through complex, natural scenes. This task is likely supported by a group of neurons in the optic lobe which respond to moving targets that subtend less than a few degrees. These Small Target Motion Detector (STMD) neurons are tuned to both target size and velocity, whilst also exhibiting facilitated responses to targets traveling along continuous trajectories. When presented with a pair of targets, some STMDs generate spiking activity that represent a competitive selection of one target, as if the alternative does not exist (i.e., selective attention). Here, we describe intracellular responses of CSTMD1 (an identified STMD) to the visual presentation of targets embedded within cluttered, natural scenes. We examine CSTMD1 response changes to target contrast, as well as a range of target and background velocities. We find that background motion affects CSTMD1 responses via the competitive selection between features within the natural scene. Here, robust discrimination of our artificially embedded “target” is limited to scenarios when its velocity is matched to, or greater than, the background velocity. Additionally, the background’s direction of motion affects discriminability, though not in the manner observed in STMDs of other flying insects. Our results highlight that CSTMD1’s competitive responses are to those features best matched to the neuron’s underlying spatiotemporal tuning, whether from the embedded target or other features in the background clutter. In many scenarios, CSTMD1 responds robustly to targets moving through cluttered scenes. However, whether this neuronal system could underlie the task of competitively selecting slow moving prey against fast-moving backgrounds remains an open question.

## Introduction

In diverse species of animals, the ability to detect prey, predators and mates within the environment is essential to survival. This task often involves the detection of small targets against highly cluttered scenes, including distracting features such as falling leaves or other animals. Numerous species have developed strategies for detecting small targets across a variety of sensory modalities, including auditory localization in bats (Arlettaz et al., 2001), the lateral line organ in squid (York et al., 2016) or visual cues in insects (Nordström and O’Carroll, 2006; Wiederman and O’Carroll, 2011), archerfish (Schuster et al., 2006) and humans (Bravo and Farid, 2004). Despite having relatively small brains and limited visual resolution (Horridge, 1978; Land, 1997), flying insects can detect and pursue small moving targets in natural scenes. This makes insects an attractive model for investigating the neuronal computations underlying these complex, sensorimotor processes.

Biological systems employ a variety of strategies to improve target tracking in clutter. These include physical adaptations, such as faster photoreceptors (Weckström and Laughlin, 1995) and more acute and sensitive subregions of the eye (Horridge, 1978; Olberg et al., 2007). Such improved spatial resolution (Hornstein et al., 2000; Burton and Laughlin, 2003) and contrast sensitivity (Gonzalez-Bellido et al., 2011) can enable the detection of smaller and dimmer targets (Straw et al., 2006; Rigosi et al., 2017). Behaviorally, insects increase catch success by fixating targets in their optical acute zones during pursuit (Olberg et al., 2007; Lin and Leonardo, 2017; Wardill et al., 2017), with dragonflies using predictive pursuit strategies (Mischiati et al., 2015; Lin and Leonardo, 2017) to intercept targets (Olberg et al., 2000). While these adaptations improve neuronal sensitivity, how these targets are discriminated from cluttered backgrounds is still poorly understood, especially in dynamic, natural environments.

Neurons tuned to small moving targets likely to underlie these behaviors have been described from several flying species (Collett, 1971; Collett and King, 1975; O’Carroll, 1993; Nordström and O’Carroll, 2006; Geurten et al., 2007; Keles and Frye, 2017). In dragonflies, Small Target Motion Detectors (STMD) respond robustly to the visual presentation of small targets (subtending less than 5 degrees) moving at any location within the neuron’s receptive field (O’Carroll, 1993). These neurons are sensitive to target contrast and are tuned to both the size and velocity of the target (Wiederman et al., 2013; O’Carroll and Wiederman, 2014).

CSTMD1 (Centrifugal Small Target Motion Detector 1), a well-studied STMD in the dragonfly *Hemicordulia tau* (Geurten et al., 2007), is an important model for investigating neural mechanisms underlying target detection, even when embedded in natural images (Wiederman and O’Carroll, 2011). CSTMD1’ receptive field includes excitatory and inhibitory hemifields on opposite sides of the visual midline. CSTMD1 exhibits predictive modulation of gain which facilitates neuronal responses to targets moving on continuous trajectories, whilst suppressing stimuli appearing at non-contiguous locations (Wiederman et al., 2017). Additionally, CSTMD1 shows selective attention, responding only to a single target when presented with a pair of alternatives, completely ignoring the second target (Wiederman and O’Carroll, 2013; Lancer et al., 2019).

Different forms of attention exist across multiple vertebrates including primates (Reynolds and Desimone, 2003) and barn owls (Mahajan and Mysore, 2018) as well as invertebrates such as bees (Morawetz and Spaethe, 2012), butterflies (Gamberale-Stille et al., 2019) and flies (van Swinderen, 2007; Jovanic et al., 2016). Selective attention refers to where multiple competing stimuli exist and one or more are preferenced while the others are suppressed. In some attentional systems weaker stimuli are amplified to maintain attention (Reynolds and Desimone, 2003) whereas in others there is an absolute encoding of the original stimulus (Chelazzi et al., 1998). CSTMD1 is in this second category and can lock-on to weakly salient targets maintaining the weakly excitatory response while ignoring high salience distractors (Lancer et al., 2019).

When confronted with targets embedded within cluttered scenes, some STMD neurons continue to respond robustly despite potential conflicting features of the background (Nordström et al., 2006; Wiederman and O’Carroll, 2011). However, these visual stimuli were limited to artificially generated backgrounds with low phase congruence (Nordström et al., 2006) or spatially constrained natural scenes without relative motion cues (Wiederman and O’Carroll, 2011). Similar studies have been conducted in the hoverfly *Eristalis*, where Target Sensitive Descending Neurons (TSDNs) respond robustly to small targets in the absence of clutter (Nicholas et al., 2018). TSDN responses were suppressed with target motion parallel to the background motion and facilitated when target motion opposed background motion (Nicholas and Nordström, 2021).

While the ability of a subset of STMDs to discriminate targets without relative motion is impressive, dragonflies operate in highly dynamic environments, where foreground (target) and background motion can vary dramatically, particularly during conspecific pursuit flights (Lin and Leonardo, 2017). How dragonfly STMD neurons respond to targets in natural scenes with varying degrees of relative motion remains unknown. Furthermore, the discovery of selective attention in STMDs raises the question of how such competitive processes interact with target-like background features inherent within natural imagery, especially since prior studies have shown that relative motion is not a prerequisite for responses to embedded targets (Nordström et al., 2006; Wiederman and O’Carroll, 2011).

Here we describe CSTMD1 responses to a small, dark, moving target superimposed on independently moving natural images. CSTMD1 exhibits reduced responses when targets are presented against visual clutter, particularly if the background speed is higher than that of the target. We show that this is not due to variable target contrast or inhibitory interactions from the background motion *per se*, but rather as the result of a competitive interplay between target and background features via selective attention. We also show the importance of CSTMD1’s velocity tuning, with the selection of faster background features (better matched to the neuron’s velocity optimum) over the slower moving foreground target.

## Methods

### Electrophysiology

Thirty-five wild-caught dragonflies (35 *Hemicordulia tau*, 33 male, two female) were immobilized with 1:1 beeswax and rosin mixture and fixed to an articulated magnetic stand with the head tilted forward to access the posterior surface. A hole was cut above the brain to gain access to the lobula and lateral midbrain, but the preparation was otherwise left with the perineural sheath and overlying haemolymph sacs intact. All dissections were performed on the left side of the animal corresponding to a right excitatory hemifield. We penetrated the sheath and recorded intracellularly using aluminosilicate micropipettes (OD = 1.00, ID = 0.58 mm), pulled on a Sutter Instruments P-97 puller and backfilled with either KCl (2 M, electrode tip resistance typically 50–150 MΩ) or 4% Lucifer Yellow solution in 0.1 M LiCl. Electrodes were placed in the medial portion of the lobula complex and stepped through the brain from posterior to anterior through the lobula complex, using a piezoelectric stepper (Marzhauser-Wetzlar PM-10, Wetzlar, Germany). Intracellular responses were digitized at 5 kHz with a 16-bit A/D converter (National Instruments) for offline analysis.

### Visual Stimuli

We presented stimuli on high-definition LCD monitors (Asus ROG Swift PG279Q 165 Hz). The animal was placed 20 cm away and centered on the visual midline with the head tilted forward such that back surface of the eye was in line with the top of the monitor (approximately 60° tilt). The display projection was distorted using OpenGL to ensure each 1° onscreen was 1° from the dragonfly’s perspective, extending 104° (−52 to 52° azimuth) by 58.5° (21 to 80° elevation from equator).

**Figure 1:**
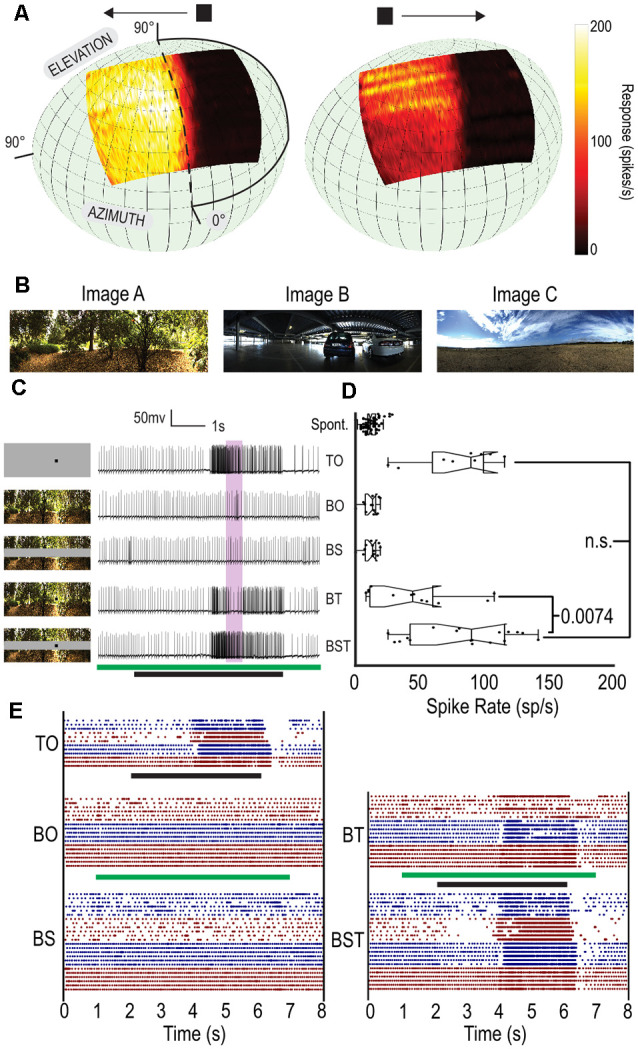
A gray strip can maintain constant, local contrast for small, moving targets. **(A)** Averaged receptive field for CSTMD1 superimposed on animal’s visual field (*n* = 11). CSTMD1 is a binocular large-field STMD with an excitatory and inhibitory hemifield, preferring targets moving away from the midline. **(B)** The three natural scenes used for experiments. **(C)** The five conditions with a corresponding example raw spike trace, Target Only (TO), Background Only (BO), Background with Strip (BS), Background with Target (BT) and Background with Strip and Target (BST). Stimulus timing shown by green (Background and Strip) and black (Target) bars. **(D)** Box plots of the five stimulus conditions. Each point represents spike rate in a 500 ms window (shown in purple in **B**), in three neurons across three dragonflies (individual trials treated independently). **(E)** Raster plots for all stationary background trials separated by condition. Black stimulus bar indicates time target is moved on the display. Green stimulus bar indicates time background is moved on the display. Color changes (red, blue) represent different cells.

Neuronal classification was performed by presenting a sequence of stimuli: a gyrated, randomly generated texel pattern (1°), gray to black and gray to white full screen flicker (White 338 cd/m^2^, Black 0.5 cd/m^2^), moving edges (up, down, left and right, 25°/s), moving bars (2° width, up, down, left and right, 25°/s) and a square-wave grating pattern (0.025 cycles/°, 6.25Hz, up, down, left and right) and a small target (1.5°) moving at 80°/s horizontally (both left and right) at 21 different elevations to map the receptive field (Figure 1A). CSTMD1 was identified based on characteristic responses to these visual stimuli including distinctive spike waveforms (Fabian and Wiederman, 2021) and unique receptive fields.

A 1 s prestimulus period of a mid-gray screen for experiments involving natural images. A post-stimulus period of 1 s was also recorded. For experiments involving repetitive stimulation of a single region, an inter-trial rest of between 15 s and 30 s was used to minimize influences from long-term adaptation. Between trials there is also some degree of variability in response strength. To assay across these factors (images and conditions), intracellular recordings are held for up to several hours, therefore we control for inter-trial variation (fatigue or adaptation) via randomization of the order of stimulus presentation.

Natural imagery used in experiments were 360° panoramas captured using a Nikon D-70 digital camera and panoramic tripod head (Brinkworth and O’Carroll, 2009). A subregion of each image was displayed to maximize vertical extent and maintain aspect ratio with the initial azimuth (referred henceforth as image phase) defined by the stimulus.

### Data Analysis and Statistics

We developed custom-written MATLAB scripts for spike counting and analysis. All statistical tests were either paired *t*-tests (with Bonferroni correction where appropriate) or n-dimensional ANOVA using tukey-kramer for multiple comparisons. All p values are reported as raw numbers in text if significant differences exist (unmarked otherwise) or as <0.0001 if sufficiently small. Box and whisker plots represent the 75th, 50th and 25th quartiles (lines) with raw data overlaid.

## Results

### Static Backgrounds

When presenting a small moving target in visual clutter the effective contrast of the target is modulated, moving over dark or bright regions. To remove this source of ambiguity, we introduced a solid gray (0.5 Weber contrast) strip over the intended trajectory of the target (similar to Nicholas et al., 2018), allowing the target to move across the field of view with constant local contrast. The strip was 11° in height and the edges of the strip were partially transparent (using a linearly declining alpha channel over 2°) to prevent the display of hard edges.

To test the efficacy of this gray-strip paradigm, we chose three natural image backgrounds (Figure 1B) to represent dense, urban and sparse environments. The panoramic background images were presented starting at one of two initial starting phases from the 360°. We tested the stimulus in five different conditions (Figure 1C): Target alone (TO) against a gray background, Background Alone (BO), Background with Strip (BS), Background with Target (BT) and Background, Strip and Target (BST). In all cases, the stationary background and strip (where present) were introduced together (Figure 1C, green bar) while the moving target (a 1.5 × 1.5° black square) was displayed 1 s after the background. The target appeared at the edge of the screen and moved horizontally at 25°/s (Figure 1C, black bar) in CSTMD1’s weakly preferred direction (left to right). Due to the excitatory and inhibitory subregions of CSTMD1’s receptive field, spiking activity elicited by the target is not apparent until halfway through the stimulus duration.

Figure 1C shows five individual spike traces from CSTMD1, one from each of the five conditions. The TO condition reveals a characteristically strong response beginning approximately half-way into the target’s appearance. In BO and BS (where no target is present) we observe limited activity over the same period. In the BT trials, we see a similar response to the TO trial, except that there is less activity part way through the target presentation. In the BST trial, we observe a similar response to the TO trial.

To examine aggregate responses, we determined spike rate (Figure 1D) over a 500 ms period starting 500 ms after the target cross the midline into excitatory receptive field (purple shaded region Figure 1C). When presented alone, the target (TO) generates strong responses in this window whereas in the BO and BS (no target present) trials the response matches the spontaneous spike rate (Figure 1C). When the background and target are presented without the strip (BT) the responses vary considerably with some low responses similar to the spontaneous spike rate. The increased variation in spike rate is expected as the local contrast of the different backgrounds varies, modulating the effective contrast of the target. With the introduction of the gray strip (BST), there was no significant difference between the TO and BST trials, whilst there was a significant difference between BT and BST trials (*p* = 0.0074, *n* = 3).

To examine these differences further, we generated raster plots for each of the individual trials separated by condition (Figure 1E). The BO and BS cases show no discernible change in response to the presentation of the background (1–7 s) except minor transient events at image onset and offset. The BT case shows increased variation with some trials exhibiting a muted response (lower overall spike-rate) due to poorer contrast and other exhibiting more variation due to the variable contrast of the image. The TO and BST cases are almost indistinguishable from one another. Thus, the gray strip paradigm is effective at eliminating the confounding effect of local, contrast variation between target and background.

### Moving Backgrounds

**Figure 2:**
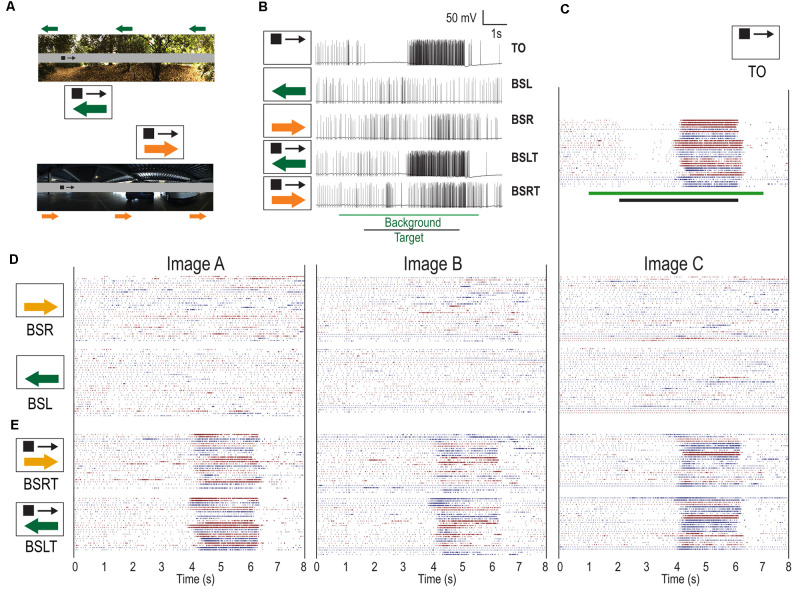
Individual responses of CSTMD1 (*n* = 12) to moving targets over moving backgrounds of either direction. **(A)** Example of stimuli with either a leftwards or rightwards moving background (green arrows) and rightwards moving target (black arrow). Corresponding pictograms are illustrated (not to scale). **(B)** Example raw traces from CSTMD1 when presented with target alone (TO), leftward moving background alone (BSL), rightward moving background alone (BSR), target with leftward background (BSLT) and target with rightward background (BSRT). Background features can both elicit activity as well as decrease responses. **(C)** Target only spike rasters (TO). Black stimulus bar indicates time target is moved on the display. Color changes represent different animals. Within any one animal, trials with the same phase are adjacent. **(D)** Responses to leftward moving backgrounds (BSL) and rightward moving backgrounds (BSR). Green stimulus bar indicates time background is moved on the display. The moving backgrounds result in sporadic responses of varying strengths. **(E)** Backgrounds moving in either direction, with a target moving in the preferred (BSLT, BSRT), are less responsive during the target epoch, compared to target alone (TO) responses.

Having demonstrated the effectiveness of the gray-strip paradigm, we examined how CSTMD1 responses are altered by moving backgrounds. We drifted the same 1.5 × 1.5° target across the gray strip at 25°/s whilst simultaneously having one of three background images moving horizontally at 15°/s (Figure 2A) presented at two different starting phases (0, 180°). To ensure differences in responses were not hidden within neuronal saturation, we chose test speeds away from the saturating, velocity optimum (60–90 deg/s). We presented five stimulus conditions (Figure 2B): Target only (TO), Leftward Background with Strip (BSL), Rightward Background with Strip (BSR), Leftward Background with Strip and Target (BSLT), Rightward Background with Strip and Target (BSRT).

Figure 2B shows individual traces measured in response to the targets and background. In the TO case, we observed strong inhibition (left hemisphere) followed by strong excitation (right hemisphere). In the BSL and BSR conditions we observed little sustained excitation or inhibition, but instead transient responses of varying strengths throughout the stimulus time course. This differs from fly TSDNs, which show consistent inhibitory responses to widefield motion (Nicholas et al., 2018). In this BSLT trial, we observed responses similar to the TO condition. In comparison, the BSRT example reveals some excitation even though the target was in CSTMD1’s inhibitory hemifield. Then, a weaker response corresponding to the period when the target was in the excitatory receptive field.

To examine these inter-trial variations more closely, we generated raster plots for each of the individual trials separated by condition (Figures 2C–E). We observed robust inhibitory and excitatory responses when the target was presented alone (Figure 2C). The variability observed in TO trials is largely due to variations in overall spike activity between animals. When the background was presented alone (BSL, BSR), responses were weaker, transient, and intermittent (Figure 2D) including less consistent inhibition in the inhibitory hemifield. These responses showed little consistency across trials, even with the same background image and background phase. In the BSLT and BSRT trials, the response to the target was still apparent in most trials but could be weaker, shorter and more variable (Figure 2E). Additionally, there are also intermittent responses when the target is not in the excitatory hemifield.

From the raster plots, we can identify where the changes are most (and least) prevalent. Most individual trials retained robust responses while the target was close to the midline of the animal (just after 4 s) with less spiking activity occurring when the target was located at a more peripheral location (5–6 s). The raster plots reveal differences in neuronal activity elicited by the different background images. Image B has the largest effect on target response (large gaps in both BSLT and BSRT). Image A has less pronounced changes, especially in the BSLT case where consistent target responses were most common. While variation of CSTMD1 activity across dragonflies is typical (Figure 1D), the strong sustained inhibition followed by sustained excitation is consistent (Figure 2C). Moreover, dragonfly Lobula Tangential Cells (LTCs), neurons sensitive to wide-field optic flow, give remarkably consistent responses to moving natural scenes, regardless of the image statistics (Evans et al., 2019). Thus, if CSTMD1 were inhibited by widefield motion in a manner similar to *Eristalis* TSDNs, we would expect to see a consistent effect of the background’s motion on CSTMD1 responses. This might appear as inhibitory or spontaneous activity in response to BSR trials and with facilitatory effects concomitant to increased relative motion between target and background in BSL trials. Instead, we see sporadic excitation of variable strength with differences between images.

We tested the differences in response between images by finding the mean spike rate in a 1 s window (4.5–5.5 s) for all BSLT and BSRT trials. We found a statistically significant difference of Image B in the BSLT case compared to both other images (A vs. B, *p* = 0.0026, B vs. C, *p* = 0.0145, 1-way ANOVA, Tukey-Kramer correction.) and no differences in the BSRT trials. Based on previous analysis of LTCs (Evans et al., 2019), we would expect Image C to be an optic flow “outlier” due to its lower image contrast. For a given speed, any inhibitory influences from an LTC would be consistent (across images). This is not observed, thus their role in CSTMD1 responses is unlikely.

**Figure 3:**
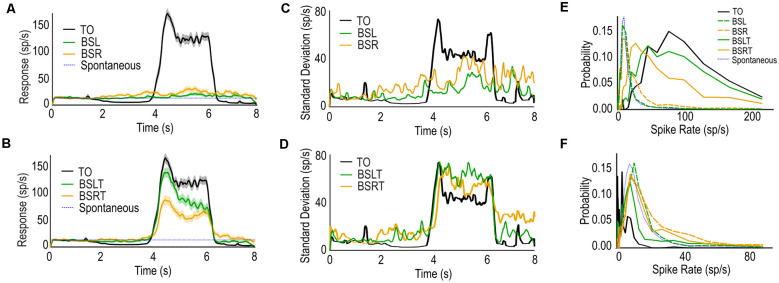
**(A)** Mean spike-rate and standard error (shaded regions) of CSTMD1 to TO, BSL, and BSR. Backgrounds alone produce modest spike rate increases. **(B)** as **(A)** for TO, BSLT, and BSRT. Targets with leftward or rightward backgrounds result in robust, but reduced spike-rates. **(C)** Standard Deviation of responses calculated over time TO, BSL, and BSR. Backgrounds alone (BSL/BSR) result in increased variation of spike rate. **(D)** as **(C)** for TO, BSLT, and BSRT (right). When the background is presented with the target (BSLT/BSRT) the variation in both the inhibitory and excitatory receptive fields are increased despite lower overall inhibition or excitation. **(E)** Weighted Histogram of Interspike Intervals of each condition over 4.25–4.75 s window (during target excitatory phase). **(F)** as **(E)** but 3.25–3.75 s window (during the target inhibitory phase).

We calculated mean spike rate as the Inverse Interspike Interval (1/ISI) across trials, averaging across different images and image phases (Figures 3A,B). The TO trials exhibit a characteristic period of inhibition (2–4 s) followed by strong excitation (4–6 s) as the target crosses the inhibitory and excitatory hemifields, respectively. When presented alone, the background (BSL and BSR) show responses above spontaneous (dotted blue, Figure 3A), stronger in the BSR case.

Background with rightwards target trials (BSRT) exhibited the least inhibition when the target was located in the inhibitory hemifield and the least excitation when in the excitatory hemifield (Figure 3B). We did not observe any facilitation compared to target alone, when target and background moved in opposing directions (BSLT, green) as observed in fly TSDNs (Nicholas and Nordström, 2021).

We further examined the variability observed in the raster plots (Figures 2C–D). Firstly, we calculated the instantaneous spike interval (ISI) by smoothing the spike times such that each spike event was replaced with the average spike time of its seven closest neighbors. This accounted for spike bursts, an additional source of variability when CSTMD1 is stimulated (Fabian and Wiederman, 2021). With bursts, the Inverse ISI oscillates around two means (burst and inter-burst) confounding variability measures. Additionally, we observe variation of the overall spiking activity across dragonflies. As a normalization factor, each Inverse ISI trial was divided by an overall excitation factor. This factor was the mean excitatory response (4.25–5.75 s) to TO trials for that animal. This normalized value was then multiplied by the mean of means across animals to return the result to spikes/s. We calculated the standard deviation in the instantaneous spike rate across trials for the five conditions (Figures 3C,D) and smoothed with a 50 ms moving average.

Figure 3C shows the standard deviation of the TO (black), BSL (green), and BSR (orange). In TO trials, we observed a small standard deviation during the prestimulus duration (0–1 s, gray screen) and the inhibitory period (2–4 s). Following this, we observe the largest variation at the start (4 s) and end (6 s) of the window when the target is within the excitatory region of CSTMD1’s receptive field. This represents variation in the onset of facilitation (Fabian et al., 2019) and interestingly also observed at the offset of the target’s presentation.

In the BSL and BSR trials we observed large variation for both background directions throughout the stimulus period, building over time. This is despite only a modest mean response (Figure 3A). We also observe that this variation continues after the offset of the stimulus (7–8 s) during which a gray screen is presented. This increased variation is likely due to post-excitatory rebounds common after stimulation of CSTMD1. In the TO cases, these post-excitatory inhibitory periods are highly consistent after stimulus offset (6 s), therefore eliciting a lower standard deviation (Figure 3A). Whereas the sporadic excitation seen in BSLT or BSRT trials will likely only induce sporadic post-excitatory inhibition.

In the BSLT and BSRT trials (Figure 3D), we observe high variation across the entire stimulus period. When the target is in the inhibitory hemifield (2–4 s), the variation is consistently higher than the TO trials. This is also true for when the target is in the excitatory hemifield (4–6 s). This is despite the mean spike rate for both conditions being lower than the TO case (Figure 3B).

We calculated a histogram of spike rates (1/ISI) for each stimulus condition over three periods of the stimulus; spontaneous period (0.25–0.75 s); target in inhibitory (3.25–3.75 s); and excitatory (4.25–4.75 s), with bins distributed logarithmically to better illustrate the differences at both high and low spike rates. In the analysis window corresponding to excitatory target responses (Figure 3E), the TO case (black) generates the highest spike rate overall. BSR (orange dashed) trials had higher spike rates over this period than BSL (green dashed) trials or spontaneous (blue dashed), reflective of CSTMD1’s weakly preferred direction (left-to-right). However, when the target is introduced, this relationship is reversed with higher spike rates for the BSLT trials (unbroken green) than the BSRT trials (unbroken orange). Thus, in background alone, rightward motion produces more excitation than leftwards (both low without a target). However, the inclusion of a target reverses this ordering, with the rightwards background (same direction as target) producing weaker responses than the opposing leftwards background.

In the analysis window corresponding to when a target may be within the inhibitory RF (Figure 3F), we see the TO cases (black) producing strong inhibition compared to the spontaneous activity. We also observe that both BSR (orange dashed) and BSRT (orange unbroken) produce the highest spike rates, stronger than spontaneous. If the background were merely inhibiting target responses, we would expect responses similar to spontaneous activity (blue dashed). Rather, CSTMD1 responses are elevated during this period.

In response to the inclusion of a background, what could cause weaker activity when the target is in the excitatory hemifield, increased responses when in the inhibitory hemifield and greater variability between trials? As previously mentioned, an inhibitory drive from a wide-field LTC neuron would not increase variability, nor induce sporadic responses such as those seen in BSL and BSR (Figure 2D). Instead, our results reveal variation in responses due to selective attention (Wiederman and O’Carroll, 2013). Natural images have been shown to have features which can excite CSTMD1 (Wiederman and O’Carroll, 2011) and the selection between the target and these background features would elicit sporadic responses observed during the BSL and BSR trials. The overall excitation observed in BSR trials (Figures 2D,E) would also be expected as this is CSTMD1’s preferred direction for the excitatory hemifield (left to right). CSTMD1 typically selected the high-contrast target and ignored features from within the background. However, when CSTMD1 selected background features rather than the target, we observe weaker responses, irrespective of whether the target is in CSTMD1’s inhibitory or excitatory hemifield.

### Contrast

**Figure 4:**
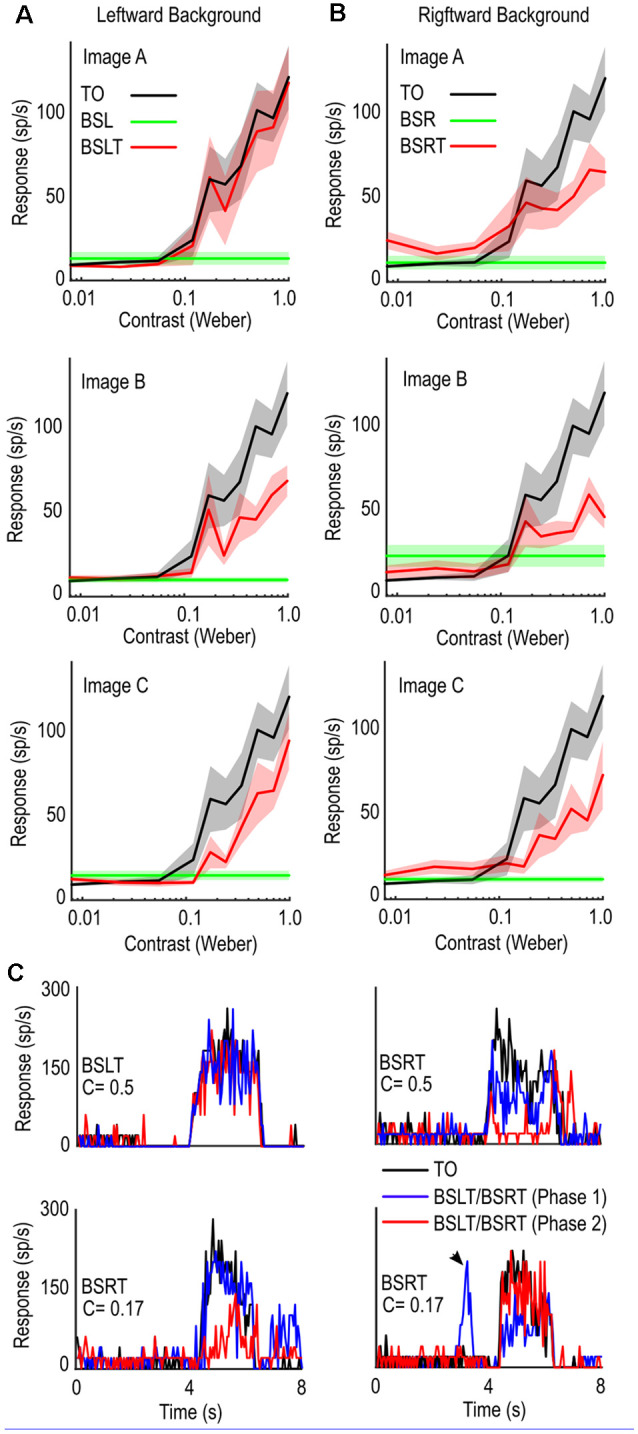
Contrast sensitivity functions in the presence of background clutter. **(A)** Comparison of target alone (black line) and target with background trial (red line) for targets of varying contrasts for leftward moving backgrounds. Introduction of background clutter causes a reduction in mean spike rate. **(B)** as **(A)** for rightward moving backgrounds. **(C)** Individual trials from three cells showing spike rate over time (Peristimulus Time Histogram). TO controls (Black) are more consistent than BSLT and BSRT trials with shifted phases (Blue, Red). Sporadic excitation can occur when target is in CSTMDs inhibitory hemifield indicating responses to background features (bottom right, black arrow).

Since background features can out-compete the high-salience target for selection, what happens when the target’s salience is lowered? To test this, we repeated previous experiment with varied target contrast. We measured spike activity while the target was within the excitatory receptive field (4.5–5.0 s) for each condition and calculated averages for each image and direction pair (Figure 4A—BSLT, Figure 4B—BSRT, *n* = 6).

The presence of background clutter reduced neuronal activity over the range of contrasts measured (Figures 4A,B). The varying degrees of shift in the BSLT and BSRT contrast sensitivity functions (across images) show that interaction between features, rather than optic flow, underlie neuronal responses (i.e., shifts would be the same to a single background speed).

As exemplars, Figure 4C shows stochastic activity from three individual neurons. Each plot shows the response to the TO (black) and two different backgrounds with target (BSLT, BSRT), where the only difference is the starting phase of the background image (red or blue line). The change in starting image phase alters when background features are within CSTMD1’s receptive field, as well as their relative position to the target. However, optic flow remains relatively constant across image phases, with minimal pattern noise expressed in LTC neurons. Here we see in some trials, the presence of background had little effect on the overall spiking activity (Figure 4C top left). In other trials (Figure 4C top right, bottom left) the starting phase of the background caused a large reduction in response at one phase (red) but not the other (blue). We also saw the introduction of sporadic responses to background features (Figure 4C bottom right, black arrow) when the target would usually generate inhibitory responses. This variability was also observed with low contrast BSLT/BSRT trials (Figure 4C bottom right, red). In this example, despite the lower contrast, we observed responses similar to the TO control.

### Velocity

**Figure 5:**
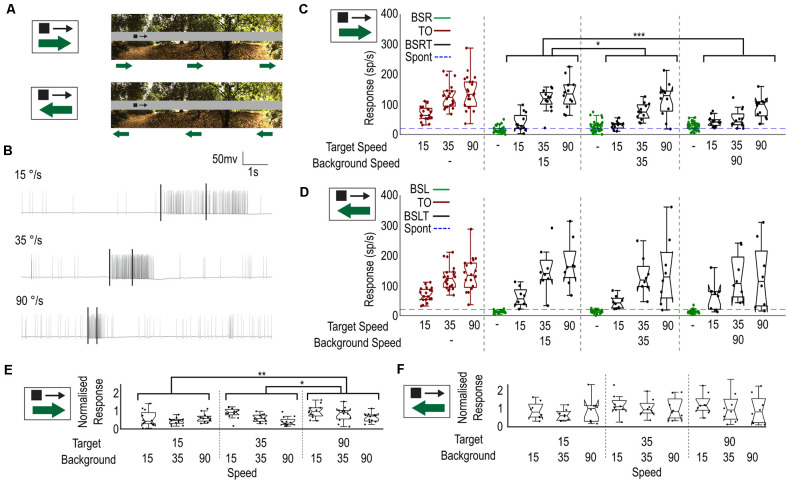
Velocity of the background influences the selection of moving targets. **(A)** Stimulus. A small black target of three chosen speeds (15°/s, 35°/s, 90°/s) moves while the background moves independently left or right at one of three chosen speeds (15°/s, 35°/s, 90°/s). **(B)** Three raw traces of varying target velocities showing the difference in response and time-course. Black bars indicate an analysis window of equal size (50°). **(C)** Box plot showing CSTMD1 responses over the analysis window, for the image conditions (target only—red, background only—green, target and background—black) across combinations of target and background velocities. This includes three target-only velocities, three background only velocities and nine target speed and background speed combinations (all in the preferred direction). Target response is more robust when the target moves as fast, or faster than the background. **(D)** as **(C)** but with the background motion opposite to the target motion. Background speed has little impact on target responses. **(E)** Normalized Target with Background (BSRT) responses for rightward moving background. Responses normalized by equivalent target-only response. **(F)** As **(E)** for leftward moving backgrounds (BSLT).

We have shown that weaker responses can be elicited in CSTMD1, when a slowly moving background (15°/s) is presented with the target. How might a faster-moving background, as experienced during rapid pursuit flights, affect neuronal responses to a fast or slow-moving target? To investigate this, we tested a single image (Figure 5A) and recorded responses to a 1.5 × 1.5° black target moving at one of three velocities (15, 35, 90°/s) against a background. The background moved at one of three different velocities (15, 35, 90°/s) in the neuron’s preferred direction (rightwards) or anti-preferred direction (leftwards), starting at one of four initial phases (0, 90, 180, 270°). We measured spiking activity in an analysis window corresponding to the moving target. As faster target velocities evoke responses over a shorter duration of time, we changed our analysis window (Figure 5B) to correspond to a period when the target moved over a constant region of space (50°) within CSTMD1’s receptive field. When the target was not present, we analyzed the corresponding period, ensuring an equivalent epoch for analysis.

Figure 5C shows the spike rate from these windows for each of fifteen conditions when the target and background direction are the same: three target-only controls for the three velocities (red); three corresponding background-only controls (green); and nine background and target speed combinations (black). The target-only (TO) trials exhibited responses matched to the underlying velocity tuning (optimum ~90°/s), as previously described (Dunbier et al., 2012). Although higher speeds produced slightly stronger responses in background-only (green) trials (one way ANOVA, *n* = 8, *p* < 0.0001), these were still very weak at all speeds compared with even the slowest target-only condition.

In BSRT trials, CSTMD1 responses strengthened as target speed increased, consistent with the inherent velocity tuning. However, at each target speed, CSTMD1 responses decreased with higher background speeds (two-way ANOVA, *n* = 8; background speed, *p* = 0.0005; target speed, *p* < 0.0001). To examine effects independent of the underlying velocity tuning, we divided all the BSRT responses by the mean of the TO trials matching their target speed (i.e., a trial with background speed of 15°/s and target speed of 35°/s was divided by the TO trial with speed 35°/s) to provide a normalized response compared to the TO alone case. After this normalization, differences we observe due to the target speed are not due to the normal velocity tuning of CSTMD1, but rather other indirect effects such as increased or decreased likelihood of selection. After normalization (Figure 5E), increases in target speed increased average responses and increases in background speed reduced average responses (two-way ANOVA, *n* = 8; background speed, *p* = 0.0026; target speed *p* = 0.0015).

How can an increased target speed cause an increase in average responses when normalized for the inherent velocity tuning? Similarly, why does increasing the background speed reduce the mean response? If features (whether target or background) are more likely to be selected when they better match the velocity tuning of CSTMD1, then we might expect that a fast background would generate more selections of “weak” background features (resulting in weaker CSTMD1 activity) and a fast target would generate more selections (stronger activity). Likewise, a slower background would generate less selections to background features (stronger activity) and a slower target is less selected (weaker activity). As there is a response difference between the well-matched target and poorly matched background features, this change in selection distribution explains the differences observed in BSRT responses.

When the background direction was reversed (BSL, BSLT, Figure 5D), we observed weaker responses from the BSL trials when compared to the BSR trials (unpaired T test, *p* < 0.0001). However, while we did observe a significant effect of target velocity on response prior to normalization (two-way ANOVA, *n* = 7, *p* < 0.0001), there was no significant effect of background speed on response (*p* > 0.5). After normalization, the effect of target speed also disappeared (two-way ANOVA, *n* = 7; background speed, *p* = 0.49; target speed *p* = 0.24, Figure 5F). Thus, with opposed target and background motion, the relative speeds are less influential. As the inhibitory hemisphere is more sensitive to leftwards motion (Bolzon et al., 2009), it is likely that the stronger competitive features are more likely to excite the inhibitory hemisphere. We have previously shown that selective attention can operate across hemispheres but is subject to different dynamics than intra-hemispheric selective attention (Lancer et al., 2022). This may explain the smaller dependence on the speed of a leftward moving background.

### Modeling Selective Attention

**Figure 6:**
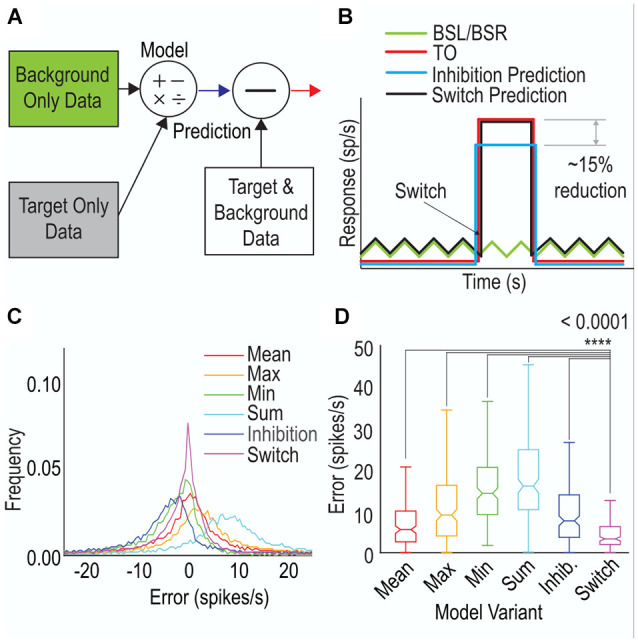
A selective attention model that includes the ability to switch, outperforms other model variants. **(A)** A schematic of the modeling approach. The target-only (TO) and background-only (BSL, BSR) data are used to predict a background with target response and this is then compared to the background with target data (mean error). **(B)** Representative illustration of a model variant; Switching (black line) generated from the corresponding TO (red line) and background-alone (green line) responses. The error is the difference between the model output and the physiological background and target responses (not shown). **(C)** Histogram of errors for the model variants Models include Mean (average of background-only and target-only responses), Max (maximum of background-only and target-only responses), Min (minimum of background-only and target-only responses), Sum (combination of background-only and target-only response and Switch (smaller error between target-only and background-only responses). **(D)** Boxplots showing mean error across each trial for each model variant. A smaller value represents a better match between model and data. The Switch model is the best match for both CSTMD1 (*p* < 0.0001, *t*-Test, Bonferroni correction).

To determine whether our selective attention hypothesis matches the observed data we developed a series of six models based on previous models used to test for selective attention (Wiederman and O’Carroll, 2013). The models were designed to predict the background with target responses (BSLT, BSRT) from different combinations of the target-alone (TO) and background-alone (BSL, BSR) physiological data (Figure 6A). We used data from our Moving Background experiments as input.

For each animal (*n* = 12), we first found the mean response of the TO trials to represent the Target-Only input. For the Background-Only input, we separated trials based on the image used, the background direction and the background starting location (phase) and then found the corresponding mean image response. This resulted in a total of twelve different Background-Only inputs (three images, two phases, two directions). Both the mean TO and set of mean BSL/BSR responses were then segmented into 5 ms bins and combined according to the rules of each model variant (six variants, details below). We then calculated an error between model output and the physiological BSLT/BSRT data (matched to the corresponding image, phase and direction).

The six variant models tested are as follows: (1) **Mean**: the average of the Target-Only or Background-Only mean responses (each in 5 ms bins); (2) **Max**: the maximum of the Target-Only or Background-Only mean responses; (3) **Min**: the minimum of the Target-Only or Background-Only mean responses; (4) **Sum**: the addition of the minimum of the Target-Only or Background-Only mean responses; (5) **Inhibition**: each Target-Only input was multiplied by a constant factor depending on the image/direction/speed group. The factor was calculated as the mean response across all BSLT/BSRT trials divided by the mean response across all TO trials; and (6) **Switch**: the smaller of the errors between either the Target-Only or Background-Only mean responses (Figure 6B).

A distribution for the errors across all model variants is shown in Figure 6C. The Switch model resulted in the smallest errors indicated by the high peaks at zero and narrow flanks. The Sum and Max models generated large over-estimates of the response while the Min model produced underestimates. We further calculated the mean absolute error for each trial (averaged across 5 ms bins) and treated each trial as an independent error (Figure 6D). The Switch model produced the smallest mean error of the six variants tested. The Switch model was significantly better than all other variants (*p* < 0.0001 *T*-Test with Boneferroni correction, in all five comparisons) indicative of a selective attention mechanism underpinning background interactions.

## Discussion

Here, we tested how CSTMD1 responds to complex scenes including high-salience targets and naturalistic background motion. We used a gray strip paradigm to eliminate variation in the target’s local contrast, allowing us to interpret long-range effects from selective attention or optic flow. We found that CSTMD1 does not simply increase or decrease responses to the target based on background motion, as might be expected by measures of relative motion. Instead, in any individual trial, CSTMD1 can remain unaffected by background motion, or selectively respond to background features (excitatory or inhibitory) rather than the high salience target. This results in an increase in inter-trial variability reflective of our previous investigations into selective attention (Wiederman and O’Carroll, 2013; Lancer et al., 2019, 2022).

These results differ markedly from other model systems including descending neurons in *Eristalis* (Nicholas et al., 2018) and some lobula small target motion detectors in *Drosophila* (Keles et al., 2020; Stadele et al., 2020). In these neurons, background motion completely suppresses responses. Additionally, *Eristalis* descending neurons presented with opposing background motion generate facilitated responses (Nicholas and Nordström, 2021). In CSTMD1, we observed intermittently weaker activity and no facilitation. This may be due to the location of CSTMD1 in the visual processing pathway, as downstream neurons may take their inputs from multiple STMDs and widefield inputs.

When dragonfly LTCs (widefield motion sensitive neurons) are presented with moving natural scenes, responses are remarkably consistent between images with differing contrast distributions and phases (Evans et al., 2019). If CSTMD1 received widefield input, we might similarly expect any differences to exhibit consistency between background images. Instead, we observed differences not only between images but even between different phases of the same image. While widefield neurons have been shown to have phase dependence (O’Carroll et al., 2011), the effect is small for large patterns. Moreover, we did not observe strong alignment of responses within individual image-phase pairs, instead observing a more stochastic response. Finally, when presented alone, the background images were net-excitatory stimuli generating sporadic responses rather than consistent suppression as is observed in flies (Nicholas et al., 2018). When two excitatory stimuli are presented together and result in net-suppression, it is indicative of a competition mechanism such as selective attention.

Our previous research found that robust responses to background features were rare (Wiederman and O’Carroll, 2011), as few background features strongly matched the finely tuned selectivity of STMD neurons. However, with the more extensive region of presented background, we found individual examples of CSTMD1 responding strongly even when a high-salience target was present in the inhibitory hemifield. These break-through excitatory responses can be attributed to background features in the excitatory hemifield out-competing a target in the inhibitory hemifield. We also observed weaker responses when the target was in the excitatory hemifield. These weaker responses were highly variable across trials, with some exhibiting no response reduction and others complete response suppression, again reflective of a selection process.

How could a low salience background feature outcompete the high salience target feature for selection? We have shown that low-contrast targets can maintain selection (a kind of “lock-on”) despite the introduction of high-contrast distracters (Lancer et al., 2019). This lock-on results in fewer observed switches. It is likely that the biasing of subsequent selection by primers that we observe is mediated by a form of ‘predictive gain modulation’ where individual target responses are increased when on predictable forward trajectories, whilst other parts of the receptive field are suppressed (Wiederman et al., 2017; Fabian et al., 2019). Similar temporal cuing behavior is also observed in flies where attention can be biased by preceding cues (Sareen et al., 2011; Koenig et al., 2016). As our backgrounds are always present in the scene prior to the target, it is possible that these features were primed and “locked-on” to. Although primed and selected background features may not exhibit strong STMD responses, the concomitant surround suppression may be sufficient to suppress distracters (enabling the “lock-on” effect described by Lancer et al., 2019). This also explains the heightened neuronal response after the target has left the field of view (Figures 3A,B) which may simply be the result of weak responses to background features.

However, if switches are rare, why are there so many trials where the target has an initially strong response followed by a far weaker response or no response at all (Figures 2, 3E)? While the lock-on effect observed reduced the number of switches, it did not eliminate them entirely, indicating a mechanism must exist to enable the switches. One possible explanation for the observed switches away from the target is that CSTMD1 might preference central targets over lateral ones. Once the target has reached a certain lateral distance, it may no longer be as salient. This reconciles with behavioral experiments where dragonflies have been shown to center targets in the middle of their field of view (Mischiati et al., 2015; Lin and Leonardo, 2017). Future experiments will investigate the locational biases that may underly competitive selection.

Our experiment varying the background velocity also reconciles with a selective attention mechanism. Faster moving targets elicit stronger responses in STMDs up to their optimal tuning at ~90°/s (Dunbier et al., 2012). This preference for fast targets is also reflected in our experiments examining predictive gain modulation, which show velocity as a key determinant of facilitation strength (Fabian et al., 2019). Thus, whether it is the target or a target-like background feature, a faster velocity (in our tested range) should result in a stronger and more facilitated underlying motion signal, improving their chance for selection. Our results reflect this, with average responses declining with faster background speeds (Figure 5C), reflecting switches away from the target to background.

How does background direction contribute to responses? When presented alone, a leftward moving background resulted in weaker activity than a rightward moving background. This reconciles with CSTMD1’s inhibitory hemifield preferring leftward motion (Bolzon et al., 2009) and CSTMD1’s excitatory hemifield preferring rightward motion. Why is it then that when presented with a target and background together, the rightward background resulted in weaker responses? We have previously shown that selection attention works across hemifields (Lancer et al., 2022). We would therefore expect that inhibition generated from background features in the inhibitory hemifield would result in weak inhibition of the response compared to the weak excitation of background features selected in the excitatory hemifield (i.e., weak inhibition is a lower response than weak excitation).

One explanation is that selective attention is separately determined in each hemifield. Communication between hemifields may only occur due to higher-order neurons such as CSTMD1 (an efferent neuron traversing the brain). In this paradigm, the target signal would only directly compete with stimuli within the same hemisphere (a process that likely involves strong inhibition). While in the excitatory hemifield, with leftward moving background, the target’s only direct competitors would be going against the preferred direction of the neuron. Thus, the target’s underlying signal strength would remain high (no inhibition from direct competitors). This strong signal would then be collated (via large interhemispheric neurons) and compete at the inter-hemisphere level where it would have a favorable chance of “winning” selection due to its high underlying salience. Thus, in the leftward moving background we would expect to see more full-strength target responses such as observed in Figure 2E.

How is the winner of this selection computationally determined? Our clutter experiments reveal that overall spike activity is not the sole determining factor. If it were, we might expect robust responses to clutter features, rivalling those of the highly salient target. Thus, the rasters in Figure 2D would only show facilitation (i.e., matching the target response when the target was the winner, and matching the stronger background feature when it was the winner). Such a facilitatory effect is observed in primate cortical cells where selection can be biased by “enhancing” the weak stimulus until it is the winner (Martinez-Trujillo and Treue, 2002; Reynolds and Desimone, 2003). Instead, we sometimes observe weak responses well matched to background features despite the presence of a target which when presented alone produces a robust strong response. Instead, one explanation is that the mechanisms determining selection are not only based on the individual STMD’s tuning for that property (e.g., size, velocity). It is possible that neurons with different tuning to larger or slower features feed into a bottom-up selective attention process, permitting switches to these less optimal stimuli. Alternatively higher-order neurons may decide arbitrarily that the high salience target is to be ignored as it fails to match another criterion for pursuit (such as lack of centrality).

While we previously showed that relative motion is not required for target detection (Wiederman and O’Carroll, 2011), here we described that relative motion reduces the likelihood of robust detection, even if the background motion is opposite to that of the target. Additionally, unlike hoverfly TSDNs, relative motion did not increase neuronal responses, even when the target was detected. This is unlike in humans where relative motion is an important cue for detection (Smeets and Brenner, 1994). Our results reveal that the most robust target detection occurs when background motion is minimized, which matches dragonfly hunting behavior (Bomphrey et al., 2016) and the saccadic flight dynamics of flies (Tammero and Dickinson, 2002). Our experiments were conducted in open-loop with a restrained animal. In contrast, closed-loop experiments in flies reveals efference copies from motor commands (Kim et al., 2015, 2017) subtract the fly’s intended movement from its widefield motion detection pathway via modulating feedback. We cannot rule out a similar effect existing in the dragonfly target detection pathway, potentially enhancing responses to targets moving against a moving background via relative motion cues.

What role might STMDs play in target selection in a behavioral context? Dragonflies have been shown to minimize the relative “slip” of a target, aiming for no relative target velocity across the eye (Mischiati et al., 2015; Lin and Leonardo, 2017). For an off-center target (i.e., the dragonfly has turned its head to fixate and is flying along an interception path), the target would have little motion, but the background scene would be moving due to the dragonfly’s ego-motion. In these scenarios, STMDs should display minimal activity, except to the background features. How might this apparent contradiction be reconciled? In close-loop pursuit, STMDs would respond robustly to target “slippage” from the midline, effectively encoding the error signal in pursuit. This matches CSTMD1’s preference for targets moving away from the midline. If STMDs are used in this capacity, the ability of the selective attention system to prioritize low-salience targets (i.e., the stationary target) would be necessary to maintain tracking.

Alternatively, the role for these STMDs may be limited to the detection phase whilst hawking, with only a minor contribution in the subsequent pursuit phase of any engagement. Our findings match the hawking behaviors of dragonflies like *Hemicordulia*, which often attempt to hover in regions where the background is clear sky. These situations maximize the contrast of potential prey and minimize the interference of motion from background distracters.

Despite these limitations, STMDs still respond robustly to targets in challenging dynamic, cluttered scenarios. Dragonflies do not always choose their engagements, with territorial or sexual conspecific encounters often commencing with the target hidden in front of cluttered background. In these circumstances, and the complex pursuit flights that follow, STMDs are still capable of generating robust responses to moving targets whilst suppressing the responses of distracting clutter. This is at odds with findings from flies, which appear to suppress target detection in the presence of moving clutter (Nicholas et al., 2018; Keles et al., 2020), perhaps making dragonflies better suited to dynamic and complex predatory pursuits.

## Data Availability Statement

The raw data supporting the conclusions of this article will be made available by the authors, without undue reservation.

## Author Contributions

BE: experimental design, data collection, data analysis and interpretation, manuscript author. DO’C and SW: experimental design, data analysis and interpretation, manuscript reviewer. JF: data collection, data analysis and interpretation, manuscript reviewer. All authors contributed to the article and approved the submitted version.

## Conflict of Interest

The authors declare that the research was conducted in the absence of any commercial or financial relationships that could be construed as a potential conflict of interest. The handling editor declared a shared affiliation with one of the authors DO’C at the time of review.

## Publisher’s Note

All claims expressed in this article are solely those of the authors and do not necessarily represent those of their affiliated organizations, or those of the publisher, the editors and the reviewers. Any product that may be evaluated in this article, or claim that may be made by its manufacturer, is not guaranteed or endorsed by the publisher.
